# Why Do Forensic Inpatient Units Have Longer Lengths of Stay? a Retrospective Cross-Sectional Analysis of Inpatient Psychiatric Rehabilitation Services in Cumbria, Northumberland, Tyne and Wear (CNTW)

**DOI:** 10.1192/bjo.2026.11619

**Published:** 2026-06-30

**Authors:** Kurchi Mitra, Chanelle Willie, Evie Newman, Neeti Sud, Patrick Keown

**Affiliations:** 1 Newcastle upon Tyne Hospitals NHS Foundation Trust, Newcastle upon Tyne, United Kingdom; 2 Newcastle University Faculty of Medical Sciences, Newcastle upon Tyne, United Kingdom; 3 School of Psychology, Newcastle University, Newcastle upon Tyne, United Kingdom; 4 Cumbria, Northumbria, Tyne and Wear NHS Foundation Trust, Newcastle upon Tyne, United Kingdom; 5 Hopewood Park Hospital, Sunderland, United Kingdom

## Abstract

**Aims:**

Psychiatric rehabilitation focusses on adults with complex psychosis and requires a whole system approach, spanning general adult and forensic services.

This report describes the clinical and demographic characteristics of inpatients in psychiatric rehabilitation services, and the different levels of care, including secure and non-secure services. Factors associated with longer lengths of stay are explored and reasons for longer lengths of stay on forensic units investigated.

**Methods:**

Demographic, clinical, and service factors were recorded on specified dates across 12 rehabilitation wards, encompassing general adult, forensic and learning disability secure rehabilitation units. Statistical analyses were performed on SPSS. Medians and non-parametric tests were used. Stepwise linear regression was performed with natural log transformation of length of stay data.

**Results:**

There were 173 patients; 137 in non-secure and 36 in forensic services. Majority were male (77%) with an average age of 45 years (range 20-72). Female patients were 5 years older on average. 12% were voluntary patients; 68% detained under civil sections and 20% detained under forensic sections. Schizophrenia was the most common diagnosis (N=107, 62%). 90% (N=155) had a diagnosis within the complex psychosis framework.

Multimorbidity of psychiatric diagnosis was the norm (total diagnoses=314, mean=1.8, median=2, range 1-5). Median length of stay was 262 days (range 7-3,309, IQR=465). Linearregression identified increasing age, number of psychiatric diagnoses, diagnosis of schizophrenia/schizoaffective/delusional disorder, being subject to a restriction order, diagnosis of autism, diagnosis of depression, and female gender as associated with longer lengths of stay. Chi-square and ANOVA testing indicated higher levels of multimorbidity, restriction orders and psychosis in forensic medium and low secure units compared to non-secure wards.

**Conclusion:**

Better treatments for complex psychosis, particularly with comorbid autism, are required. Increasing age and, to a lesser extent, female gender were identified as impacting on treatment outcome. Medium and low secure units had the highest rates of schizophrenia and restriction orders, as well as high rates of multimorbidity. Learning disability secure rehabilitation units had the oldest average age and the highest rate of multimorbidity. Inpatient typologies need to be expanded to include medium secure rehabilitation units and interface with learning disability services.

